# Perception and Attitude of College Students toward Cosmetic Rhinoplasty 

**DOI:** 10.22038/IJORL.2023.70373.3389

**Published:** 2023-07

**Authors:** Kamal Alshami, Raghad Alsaati

**Affiliations:** 1 *Dr Soliman Fakeeh hospital ENT department, Jeddah, Saudi Arabia.*; 2 *Faculty of Medicine and Surgery Program, Fakeeh College for Medical Sciences, Jeddah, Saudi Arabia.*

**Keywords:** Medical students, Understanding, Surgical rhinoplasty, Situation, Saudi Arabia

## Abstract

**Introduction:**

Rhinoplasty, a surgery that reshapes the nose to increase facial beauty or to restore the nasal function after a physical defect, is the most popular plastic surgery worldwide and second most common in Saudi Arabia. Regardless of how common this procedure is done in Saudi Arabia, there is a huge lack of studies on how medical students feels and acts towards it. Thus, this study aimed to assess the perception and attitude of cosmetic rhinoplasty among Fakeeh college students in Jeddah, Saudi Arabia.

**Materials and Methods:**

A validated questionnaire about participants desire to rhinoplasty was randomly distributed among all Fakeeh College students in a simple random technique.

**Results:**

306 participants completed the questionnaire Most responders (60.1%) were happy with their noses. A significant association was noted between gender and desire to undergo rhinoplasty (P=0.034). Also, there is a significant relationship between the participant’s desire for rhinoplasty and its cost (P = 0.034).

**Conclusion:**

This study showed although rhinoplasty is socially accepted in Saudi Arabia. there is a low desire in college students toward performing rhinoplasty. Factors like cost of surgery or gender affect people’s willingness to do rhinoplasty.

## Introduction

Rhinoplasty is the most popular type of plastic surgery that basically reshapes the nose to increase facial beauty or to restore the nasal function after a physical defect, such as birth disorders, trauma, burns, developmental abnormalities, infections, tumors, preference and self-choice (1,2). 

Nose appearance and length can affect self-perception in both genders because the nose is located in the center of the face. 

Thus, rhinoplasty is becoming more widely used (3). Rhinoplasty has complications which include as in any surgical procedure, which include complications in anesthesia, unsatisfactory appearance and deformity, septal perforation and hematoma, epistaxis, infection and worsening in nasal functions or in the shape rather than improvement (4,5).

Rhinoplasty is considered the fifth most commonly performed plastic surgery type and the second most common in Saudi Arabia (2). 

Regardless of how common this procedure is done in Saudi Arabia, studies on how the population feels and acts toward it particularly medical students who are more exposed and knowledge regarding rhinoplasty, are lacking. Thus, this study aimed to assess the perception and attitude of cosmetic rhinoplasty among Fakeeh college students in Jeddah, Saudi Arabia.

## Materials and Methods

This study was approved by the Fakeeh College for Medical Sciences (FCMS) committee. A cross sectional study was conducted randomly among Fakeeh College students in Jeddah, Saudi Arabia from.

Fakeeh students in all academic years of four departments (Medicine, MLS, Nursing, and Pharma D) were included, and an electronic self-administered questionnaire was distributed online from February to June 2022. 

The sample size was 292, which was calculated by a Raosoft calculator. 

In this study, we used an online questionnaire that was validated, and the following sections were included:

First section: - Demographic data: (Gender, age, discipline, academic year)

Second section: Attitude of the sample, which included 12 questions: The answers for each question are as follows: 

1. Feeling about Your nose? Happy, Not happy, Do not care.

2. Any previous cosmetic rhinoplasty? Yes, No.

3. Are rhinoplasty necessary? Yes, No, Not sure.

4. Any family history of cosmetic intervention? Yes, No.

5. Has a friend undergone cosmetic surgery? Yes, No.

6. Are you embarrassed of cosmetic procedures if anybody noticed it? Yes, No. 

7. Would you undergo cosmetic procedures if your friend tell you to do so? Yes, No, Uncertain. 

8. If cosmetic surgeries were done for free, would you go for any cosmetic 

surgery? Yes, No, Not sure.

9. If you were cognizant that somebody underwent cosmetic surgery, does this influence your relationship with him? Yes, No, Not sure.

10. Is It socially accepted in Jeddah? Yes, No, Not sure. 

11. If (10) yes, to what extant is it acceptable? Widely, Averagely, Not acceptable. 

Data collection technique: A validated questionnaire was randomly distributed among all Fakeeh College students using a simple random technique. 

Data analysis: Data received from the online survey were entered into Microsoft Office Excel 2016 and analyzed using the Statistical Package for Social Sciences (SPSS) version 25. A P-value of<0.05 was considered significant.

## Results

 Among the 306 student participants, (247 (80.7%)) were females and (59 (19.3%)) males. Most of the students (298 (97.4%)) were aged 18 – 25 years. Most of the students were Medicine (201 (65.7%)) (Table 1).

 The percentage of students with a history of previous rhinoplasty was 10.8%. 

 Most of the students feel happy regarding their noses (184 (60.1%)). A total of (123 (40.2%)) chose no when we asked if rhinoplasty is necessary. Ninety-three (30.4%) had a family history of cosmetic intervention. Majority (199 (65.0%)) felt that rhinoplasty is socially accepted in Jeddah (Table 2). 

**Table 1:** Demographic characteristics of respondents

**Characteristics**		**N (%) **
Gender	Female	247 (80.7%)
	Male	59 (19.3%)
Age groups	below 18	1 (0.3%)
	18-25	298 (97.4%)
	Above 25	7 (2.3%)
Specialty	Medicine	201 (65.7%)
	MLS	20 (6.5%)
	Nursing	59 (19.3%)
	Pharm D	26 (8.5%)
Academic year	1st year	15 (4.9%)
	2nd year	80 (26.1%)
	3rd year	92 (30.1%)
	4th year	52 (17.0%)
	5th year	40 (13.1%)
	6th year	27 (8.8%)

**Table 2:** Attitudes of Fakeeh medical students towards cosmetic rhinoplasty

**Question**	**Attitude**	**N (%)**
Your feeling regarding your nose	Happy	184 (60.1%)
	Not happy	59 (19.3%)
	Don’t care	63 (20.6%)
Any previous cosmetic rhinoplasty?	Yes	33 (10.8%)
	No	273 (89.2%)
Is rhinoplasty necessary?	Yes	71 (23.2%)
	No	123 (40.2%)
	Not sure	112 (36.6%)
Any family history of cosmetic intervention?	Yes	93 (30.4%)
	No	213 (69.6%)
Has a friend undergone cosmetic surgery?	Yes	126 (41.2%)
	No	180 (58.8%)
Are you embarrassed of cosmetic procedures if anybody noticed it?	Yes	59 (19.3%)
	No	247 (80.7%)
Would you undergo cosmetic procedure if your friends tell you to do so?	Yes	151 (49.3%)
	No	64 (20.9%)
	Uncertain	91 (29.7%)
If cosmetic surgeries were done for free, would you go for any cosmetic surgery?	Yes	106 (34.6%)
	No	138 (45.1%)
	Not sure	62 (20.3%)
If you were cognizant that somebody underwent cosmetic surgery, does this influences your relationship with him?	Yes	27 (8.8%)
	No	257 (84.0%)
	Not sure	22 (7.2%)
Is it socially accepted in Jeddah?	Yes	199 (65.0%)
	No	22 (7.2%)
	Not sure	85 (27.8%)
If (10) yes, to what extant is it acceptable?	Widely	88 (28.8%)
	Averagely	124 (40.5%)
	Not acceptable	94 (30.7%)

 A significant association was noted regarding gender and undergoing cosmetic surgery affecting relationships with a P value = 0.029 (Table 3). A significant association regarding specialty and undergoing cosmetic surgeries if performed for free, with a P value = 0.034 and if it is socially accepted to undergo rhinoplasty in Jeddah with a P value = 0.043 (Table 4).

**Table 3:** Attitudes of gender towards cosmetic rhinoplasty

**Question**	**Response**	**Male**	**Female**	**Total**	**P-value**
1. Your feeling regarding your nose		**N (%)**			
	Happy	35 (11.4%)	149 (48.7%)	184 (60.1%)	0.483
	Not happy	9 (2.9%)	50 (16.3%)	59 (19.3%)	
	Don’t care	15 (4.9%)	48 (15.7%)	63 (20.6%)	
2. Any previous cosmetic rhinoplasty?	Yes	8 (2.6%)	25 (8.2%)	33 (10.8%)	0.595
	No	51 (16.7%)	222 (72.5%)	273 (89.2%)	
3. Is rhinoplasty necessary?	Yes	12 (3.9%)	59 (19.3%)	71 (23.2%)	0.616
	No	27 (8.8%)	96 (31.4%)	123 (40.2%)	
	Not sure	20 (6.5%)	92 (30.1%)	112 (36.6%)	
4. Any family history of cosmetic intervention?	Yes	17 (5.6%)	76 (24.8%)	93 (30.4%)	0.891
	No	42 (13.7%)	171 (55.9%)	213 (69.6%)	
5. Has a friend undergone cosmetic surgery?	Yes	25 (8.2%)	101 (33.0%)	126 (41.2%)	0.951
	No	34 (11.1%)	146 (47.7%)	180 (58.8%)	
6. Are you embarrassed of cosmetic procedures if anybody noticed it?	Yes	12 (3.9%)	47 (15.4%)	59 (19.3%)	0.963
	No	47 (15.4%)	200 (65.4%)	247 (80.7%)	
7. Would you undergo cosmetic procedure if your friends tell you to do so?	Yes	24 (7.8%)	127 (41.5%)	151 (49.3%)	0.061
	No	10 (3.3%)	54 (17.6%)	64 (20.9%)	
	Uncertain	25 (8.2%)	66 (21.6%)	91 (29.7%)	
8. If cosmetic surgeries were done for free, would you go for any cosmetic surgery?	Yes	16 (5.2%)	90 (29.4%)	106 (34.6%)	0.351
	No	31 (10.1%)	107 (35.0%)	138 (45.1%)	
	Not sure	12 (3.9%)	50 (16.3%)	62 (20.3%)	
9. If you were cognizant that somebody underwent cosmetic surgery, does this influences your relationship with him?	Yes	8 (2.6%)	19 (6.2%)	27 (8.8%)	0.029*
	No	43 (14.1%)	214 (69.9%)	257 (84.0%)	
	Not sure	8 (2.6%)	14 (4.6%)	22 (7.2%)	
10. Is it socially accepted in Jeddah?	Yes	33 (10.8%)	166 (54.2%)	199 (65.0%)	0.244
	No	6 (2.0%)	16 (5.2%)	22 (7.2%)	
	Not sure	20 (6.5%)	65 (21.2%)	85 (27.8%)	
11. If (10) yes, to what extant is it acceptable?	Widely	12 (3.9%)	76 (24.8%)	88 (28.8%)	0.039*
	Averagely	21 (6.9%)	103 (33.7%)	124 (40.5%)	
	Not acceptable	26 (8.5%)	68 (22.2%)	94 (30.7%)	

**Table 4:** Attitudes based on specialty toward cosmetic rhinoplasty

**Question**	**Response**	**MBBS**	**MLS**	**Nursing**	**Pharma D**	**P-value**
1. Your feeling regarding your nose		N (%)				
	Happy	122 (39.9%)	11 (3.6%)	32 (10.5%)	19 (6.2%)	0.499
	Not happy	42 (13.7%)	4 (1.3%)	10 (3.3%)	3 (1.0%)	
	Don’t care	37 (12.1%)	5 (1.6%)	17 (5.6%)	4 (1.3%)	
2. Any previous cosmetic rhinoplasty?	Yes	17 (5.6%)	3 (1.0%)	10 (3.3%)	3 (1.0%)	0.278
	No	184 (60.1%)	17 (5.6%)	49 (16.0%)	23 (7.5%)	
3. Is rhinoplasty necessary?	Yes	43 (14.1%)	7 (2.3%)	15 (4.9%)	6 (2.0%)	0.307
	No	89 (29.1%)	8 (2.6%)	17 (5.6%)	9 (2.9%)	
	Not sure	69 (22.5%)	5 (1.6%)	27 (8.8%)	11 (3.6%)	
4. Any family history of cosmetic intervention?	Yes	63 (20.6%)	6 (2.0%)	16 (5.2%)	8 (2.6%)	0.942
	No	138 (45.1%)	14 (4.6%)	43 (14.1%)	18 (5.9%)	
5. Has a friend undergone cosmetic surgery?	Yes	86 (28.1%)	9 (2.9%)	20 (6.5%)	11 (3.6%)	0.650
	No	115 (37.6%)	11 (3.6%)	39 (12.7%)	15 (4.9%)	
6. Are you embarrassed of cosmetic procedures if anybody noticed it?	Yes	31 (10.1%)	7 (2.3%)	15 (4.9%)	6 (2.0%)	0.079
	No	170 (55.6%)	13 (4.2%)	44 (14.4%)	20 (6.5%)	
7. Would you undergo cosmetic procedure if your friends tell you to do so?	Yes	109 (35.6%)	6 (2.0%)	26 (8.5%)	10 (3.3%)	0.139
	No	38 (12.4%)	8 (2.6%)	13 (4.2%)	5 (1.6%)	
	Uncertain	54 (17.6%)	6 (2.0%)	20 (6.5%)	11 (3.6%)	
8. If cosmetic surgeries were done for free, would you go for any cosmetic surgery?	Yes	73 (23.9%)	7 (2.3%)	24 (7.8%)	2 (0.7%)	0.034*
	No	93 (30.4%)	9 (2.9%)	19 (6.2%)	17 (5.6%)	
	Not sure	35 (11.4%)	4 (1.3%)	16 (5.2%)	7 (2.3%)	
9. If you were cognizant that somebody underwent cosmetic surgery, does this influences your relationship with him?	Yes	14 (4.6%)	2 (0.7%)	7 (2.3%)	4 (1.3%)	0.430
	No	173 (56.6%)	46 (15.0%)	46 (15.0%)	22 (7.2%)	
	Not sure	14 (4.6%)	6 (2.0%)	6 (2.0%)	0(0.0%)	
10. Is it socially accepted in Jeddah?	Yes	141 (46.1%)	9 (2.9%)	35 (11.4%)	14 (4.6%)	0.043*
	No	10 (3.3%)	1 (0.3%)	8 (2.6%)	3 (1.0%)	
	Not sure	50 (16.3%)	10 (3.3%)	16 (5.2%)	9 (2.9%)	
11. If (10) yes, to what extant is it acceptable?	Widely	67 (21.9%)	3 (1.0%)	13 (4.2%)	5 (1.6%)	0.074
	Averagely	76 (24.8%)	7 (2.3%)	31 (10.1%)	10 (3.3%)	
	Not acceptable	58 (19.0%)	10 (3.3%)	15 (4.9%)	11 (3.6%)	

## Discussion

This study aimed to assess the perception and attitude of Fakeeh students towards cosmetic rhinoplasty.

The percentage of students whom practice rhinoplasty is (10.8%)according to the results and a similar result as that of our study was revealed at Al-Nahrain University (10.5%), (6) unlike a study performed at the Massachusetts General Hospital of USA. Among female undergraduate students in a university in the northeast (2.7%), had undergone a cosmetic surgical procedure (7). 

Furthermore, a study was done among medical students in Al-Taif University and the results revealed that none of the participants had underwent plastic surgery in general before (8), which could mean a higher awareness of cosmetic procedures among medical students in Massachusetts and Al-Taif University than those in our study and in Al-Nahrain university. Regarding weather a person has a relative or friend who had underwent a rhinoplasty, (30.4%) had a family history of rhinoplasty, and (41.2%) had friends’ history of rhinoplasty as a total of (71.6%), quite similar to Al-Nahrain which is (15%) individuals who had a family history of rhinoplasty, and (51%) of friends’ history (6). Additionally, a study in Riyadh had a total of (54.1%) participants who had relatives or friends with previous rhinoplasty (9). 

A very different result in Al-Taif University study revealed only (5.9%) of the participants who had a relative who underwent a cosmetic procedure (8), which might mean the high prevalence and knowledge of this procedure among the participants in our study and those in the studies, however, the opposite is true for Al-Taif study.

Regarding satisfaction (60.1%), of the participants were happy regarding their noses, which was higher than the results of a study performed among high school students in Iran, which was (47%) (4), and (48%) of the participants in Al-Nahrain study were also happy (6), which could explain the maturity of a person when in college as the individual develops higher self-esteem and thinks realistically in our study in Saudi Arabia and Iraq. however, among Iran young students, this could explain that their culture is more evident on the population and in the appearance of people. In addition, the age of the population was studied is younger than that in our study, which explains the developing in maturity that did not provide an extreme level of good self-esteem. The minority of the participants (23.2%) thinks that rhinoplasty is necessary, which is similar to that of Al-Nahrain study (28.5%), and that could be due to the high awareness of rhinoplasty procedures in both populations and the similarity in the cultures of Iraq and our country (6). 

An opposite to the study was performed among healthcare workers in Nigeria with (65.3%) of the respondents who considered cosmetic surgery necessary.(10) and Regarding the supportiveness to a friend who would undergo a cosmetic surgery, our study had a (49.3%) of the participants in our study agreed to this, which was a little less than the (60.1%) of Nigerian healthcare workers studied (10), which confirms the difference in our culture of ours and that of the Nigerians’ in addition to the difference in religion as in Muslim countries, rhinoplasty is discouraged, contrary to other countries whose religion might be different than Islam such as in Nigeria. 

The majority of the participants in this study (80.7%) was not embarrassed to undergo cosmetic procedures similar to those of Al-Nahrain study which was (70.5%) (6).

Increased awareness in regarding complications, real indications regarding rhinoplasty, and the idea that it will possibly be the best decision for everyone exists in Saudi Arabia. Regarding acceptability in the country, (40.5%) of the participants in our study think that rhinoplasty is averagely acceptable; however in a Nigeria study, only (30.0%) of the participants think that rhinoplasty is averagely acceptable (10). It might be a bit higher in our study just because our sample was bigger than theirs. Conversely, a study was done among medical students in Al-Taif University in a city near Jeddah, and (61.8%) of the participants who disagreed that cosmetic surgery is accepted socially (8), which could explain the slightly different culture of Al-Taif City; more strict in their religion. 

A total of (34.6%) the participants in our study participants would undergo cosmetic surgery if it was performed for free, which was similar to the results of Al-Nahrain study (27.5%) (6).

Furthermore, a study was done among the general population in Riyadh which had a result of (24.8%) (9). The study on the Nigerian healthcare workers has a similar result (20.2%) (10), Which might be due to the idea that the surgery it is not necessary and only a very specific situation is will make their decision worth it, such as rhinoplasty performed for free. 

Regarding gender, no significant difference in undergoing rhinoplasty was noticed similar to the results of the Iraq study (6). 

Conversely, a significant association was noted in this study regarding gender and weather knowing somebody underwent cosmetic surgery would have an effect on their relationship (p value 0.029)similar to that of Al-Nahrain study, which also had a significant association (p value 0.002 2) (6), and that of the Swami V study (11). 

## Conclusion

 This study aimed to assess the perception and attitude of Fakeeh students towards cosmetic rhinoplasty. the results revealed that the percentage of participants who underwent rhinoplasty was (10.8%), and most students (60.1%) were happy regarding their noses, (40%) disagreed on the necessity of rhinoplasty, approximately (30%) had a family history of rhinoplasty, the majority feel that rhinoplasty is socially accepted in Jeddah, gender had significance toward the effect on the relationship with someone who underwent rhinoplasty with a P value = 0.029. Another significant was found regarding specialty and undergoing cosmetic procedures for free with a P value = 0.034. 

### Limitation

 Our sample size has limited our study in such that we required more males to be included, as females are the majority at Fakeeh College. 

## References

[1] Rastmanesh R, Gluck ME, Shadman Z (2009). Comparison of body dissatisfaction and cosmetic rhinoplasty with levels of veil practicing in Islamic women. Int J Eat Disord..

[2] ISAPS Global Statistics. 2021;2020–2.

[3] Piromchai P, Suetrong S, Arunpongpaisal S (2011). Psychological status in patients seeking rhinoplasty. Clin Med Insights Ear, Nose Throat..

[4] Arabi Mianroodi A, Eslami M, Khanjani N (2012). Interest in Rhinoplasty and Awareness about its Postoperative Complications Among Female high School Students. Iran J Otorhinolaryngol [Internet]..

[5] Reid AJ, Malone PSC (2008). Plastic surgery in the press. J Plast Reconstr Aesthet Surg..

[6] Hussain NA, Kadhem QI, Hussain AMA (2020). The practicing and attitude of some medical students at Al-Nahrain college of medicine towards cosmetic rhinoplasty. Medico-Legal Updat..

[7] Delinsky SS (2005). Cosmetic surgery: A common and accepted form of self-improvement?. J Appl Soc Psychol..

[8] Hammadi HA, El-Shereef EA (2017). Study of knowledge, attitude and practices of plastic surgery among females students at faculty of education, Taif University, Saudi Arabia. Am J Public Heal Res..

[9] Almohanna S, Alswidan R, Alarfaj A, Subhan Y (2014). The Number and Awareness of Rhinoplasty and People Preferences of the Shape of the Nose.

[10] Adedeji OA, Oseni GO, Olaitan PB (2014). Awareness and Attitude of Healthcare Workers to Cosmetic Surgery in Osogbo, Nigeria. Gasparini G, editor. Surg Res Pract [Internet]..

[11] Swami V, Chamorro-Premuzic T, Bridges S, Furnham A (2009). Acceptance of cosmetic surgery: Personality and individual difference predictors. Body Image [Internet]..

